# Analysis of mutations in mitochondrial transfer RNA genes and the maternal inheritance of polycystic ovary syndrome

**DOI:** 10.3389/fendo.2025.1509791

**Published:** 2025-02-18

**Authors:** Tanzeela Nawaz, Tahira Awan, Humaira Zahoor, Romana Gul, Shaheen Bibi, Aziz Uddin, Ghulam Abbas, Sajid Ul Ghafoor, Sefealem Assefa Belay, Abdur Rehman, Xing-Guo Li, Saadia Tabassum

**Affiliations:** ^1^ Department of Zoology, Hazara University Mansehra, Khyber Pakhtunkhwa, Pakistan; ^2^ Department of Biotechnology and Genetic Engineering, Hazara University Mansehra, Khyber Pakhtunkhwa, Pakistan; ^3^ Department of Biotechnology, University of Agriculture, Dera Ismail Khan, Pakistan; ^4^ Graduate Institute of Biomedical Sciences, China Medical University, Taichung, Taiwan; ^5^ Institute of Biochemistry and Molecular Biology, China Medical University, Taichung, Taiwan; ^6^ Research Center for Cancer Biology, China Medical University, Taichung, Taiwan; ^7^ Cancer Biology and Precision Therapeutics Center, China Medical University, Taichung, Taiwan; ^8^ Department of Biological Science and Technology, China Medical University, Taichung, Taiwan; ^9^ PhD Program for Aging College of Medicine, China Medical University, Taichung, Taiwan

**Keywords:** mitochondrial tRNA genes, mutations, PCOS, genome sequence analysis, pathogenicity

## Abstract

**Introduction:**

Polycystic ovary syndrome (PCOS) is the most common endocrine disorder in women of reproductive age. Despite the escalating global prevalence, there is currently no definitive predisposition test available for this condition. Among the genetic causes, variations in the mitochondrial DNA (mtDNA) are increasingly recognized as a crucial contributor to the development of PCOS. However, cross-ethnic analysis of these mutations is lacking. To fill in this gap, our objective is to identify new maternal genetic risk factors associated with PCOS by investigating the mitochondrial transfer RNA (mt-tRNA) genes in PCOS patients from Pakistan and to compare these mutations to those in patients from other ethnic groups.

**Methods:**

DNA was extracted from saliva samples of patients. Primers were designed for the amplification of all of 22 mt-tRNA genes, and PCR was employed under defined conditions. Subsequently, Sanger sequencing was employed to decipher the sequences of mt-tRNA genes. Following sequencing, mt-tRNA genes underwent mutation analysis. Finally, we utilized MitoTIP (Mitochondrial tRNA Informatics Predictor) to identify variations in mt-tRNA genes.

**Results:**

In a cohort of 64 Pakistani patients with PCOS, our analysis unveiled eight variants in five mt-tRNA genes including MT-TH, MT-TL2, MT-TS1, MT-TS2, and MT-TT genes. All of these variants have not been previously reported in PCOS except one we have recently identified in a Pakistani patient with PCOS. Interestingly, most of these mt-tRNA genes carry variants found in patients with PCOS across distinct ethnic groups. Furthermore, these mutations occurred in highly conserved nucleotides of tRNA, essential for ensuring the stability and biochemical functionality of mt-tRNA. Finally, the pathogenic potential of these variations was assessed by *in silico* analysis. The pathogenicity prediction of these variants suggests their potential impact on mitochondrial dysfunction that was responsible for the clinical phenotypes of PCOS.

**Conclusion:**

Our study identified novel variations in mt-tRNA genes in Pakistani women with PCOS. To our knowledge, this is the first report comparing mutations of mt-tRNA genes in PCOS patients across different ethnic groups. Our data revealed common mt-tRNA genes carrying PCOS-associated mutations that may be specific to certain ethnic populations. Together, our work provides new insights into the role of mt-tRNA genes in mitochondrial dysfunction underlying the pathophysiology of PCOS, highlighting mt-tRNA mutations as potential factors for future predisposition tests and more effective therapies for this globally prevalent condition.

## 1 Introduction

Polycystic ovary syndrome (PCOS) is the most common endocrine disease affecting up to 15% of reproductive-age women worldwide (1). This highly heritable, complex genetic disorder is characterized by a variable constellation of reproductive and metabolic abnormalities, contributing to the most cases of anovulatory infertility and type 2 diabetes mellitus (T2D) in young women (1). Clinically, the National Institutes of Health (NIH) criteria (2) and the Rotterdam criteria (3, 4), the commonly used diagnostic criteria for PCOS, are based on the presence of at least two of three phenotypes: hyperandrogenism (HA), chronic oligo/anovulation or ovulatory dysfunction (OD), and polycystic ovarian morphology (PCOM) (2–4). Notably, the selection of PCOS patients is currently described in the modified Rotterdam’s criteria published in 2023 (5), which also includes the elevated testosterone and free testosterone levels apart from the previously cited criteria. Despite these substantial morbidities and significant progress in diagnostic criteria for PCOS, the underlying mechanisms accounting for the etiology of PCOS remain poorly understood, and the prevalence of PCOS continues to rise (1).

Beyond impacting fertility, individuals with PCOS have an elevated likelihood of developing obesity, insulin resistance, and metabolic disorders, all of which are interconnected with mitochondrial dysfunction (6). Mitochondria, organelles responsible for energy production, is the main source of cellular ROS (reactive oxygen species), and thus may lead to oxidative stress damage. Therefore, mitochondria-generated oxygen stress has been recognized as a crucial factor of PCOS etiology (6). Intriguingly, mutations in mtDNA have been identified in PCOS patients and they may likely play an important role in PCOS etiology and pathogenesis, even though their causative role in PCOS requires further investigation. Up to now, 33 PCOS-related mtDNA mutations have been found in patients with PCOS. Among these mtDNA mutations, the majority of mutations (20 out of 33) were identified in the D-loop regulatory region, suggesting the critical role for D-loop in mitochondria function (7). Intriguingly, 12 mutations have been found in eight mt-tRNA genes, highlighting the potential pathological role of mt-tRNA in PCOS (7).

Accumulating evidence has implicated mutations in mt-tRNA genes as important contributors to PCOS. Mutations in mt-tRNA genes in PCOS patients were first reported in 2012 (8). In this study, six variants in mt-tRNA genes were identified in a cohort of 57 Chinese Han women with PCOS. Importantly, these variants occurred at highly conserved nucleotides of the corresponding mt-tRNAs, suggesting the potential impact of these mutations on the stability and biochemical functions of mt-tRNAs (9). Further studies from the same research team revealed more mutations in mt-tRNA genes from 160 PCOS patients and 80 healthy subjects (10–13). It was proposed that mutations in mt-tRNA genes may lead to the failure of mt-tRNA metabolism, resulting in mitochondrial dysfunction that may underlie the clinical phenotypes of PCOS. These alterations in mt-tRNA may give rise to modifications in both the structure and functionality of mitochondrial RNA, which may lead to the destabilization of the tertiary structure of mt-tRNAs, alterations in RNA precursor processing, deletion of nucleotide modifications, and inadequate aminoacylation (14). In support of the potential role of mt-tRNA in PCOS pathogenesis, a recent study in human subjects identified 14 variants in seven mt-tRNA genes in a second ethnic group in Southern India (15). Furthermore, a recent transgenic mouse model demonstrated metabolic syndrome-like phenotypes in animals carrying a mutation orthologous to human pathological A3302G mutation in MT-TL1, mt-tRNA^Leu(UUR)^, found in patients with PCOS and other mitochondrial disease, providing molecular basis for understanding mt-tRNA mutation-mediated mitochondrial disorders, such as PCOS (16).

So far the mutations in mt-tRNA genes identified in PCOS patients have originated from only two ethnic groups (Chinese Han and South Indian). Given that PCOS, as a highly heterogenic syndrome, may present variable manifestations in different ethnic and age groups (17), however, the information of mt-tRNA mutation analysis from other ethnic groups is still missing. Additionally, accumulating evidence suggests the presence of potential hot-spot regions for PCOS-related mtDNA mutations, such as the D-loop regulatory region (7). Due to the lack of cross-ethnic analysis of mt-tRNA genes in PCOS patients, it remains unclear whether there are any hot-spot mt-tRNA genes that may be associated with the development of PCOS. Importantly, our group, to our knowledge, for the first time, has recently reported the presence of one novel mutation, 12308A >G in MT-TL2 in a Pakistani patient with PCOS (18). Pathogenicity analysis further indicated that this variant was likely to be associated with benign cysts, one of the key phenotypes of PCOS (18). To further understand the role of mt-tRNA genes in PCOS cross different ethnic groups, the current study was designed to screen all of the 22 mt-tRNA genes in a cohort of 64 PCOS patients and 30 healthy individuals from 30 Pakistani families, and to predict, *in silico*, the resultant variations for pathogenicity.

## 2 Materials and methods

### 2.1 Ethical statement

The experimental procedures were approved by the Ethical Committee of the Institution and Board of Advanced Studies and Research at Hazara University, Mansehra (21300), Pakistan under notification number F.No.73/HU/ORIC/IBC/2017/400.

### 2.2 Study design

Our recent study identified a variation in MT-TL2 gene in three Pakistan patients with PCOS, which is the first report on the mutations of mt-tRNA genes in Pakistan patients with PCOS (18). In the current case control study, we recruited 64 Pakistan patients diagnosed with PCOS, as well as 30 healthy women as the control group, from 30 Pakistan families. Following DNA extraction from saliva samples, Sanger sequencing was employed to decipher the sequences of mt-tRNA genes. The pathogenicity of mutations in mt-tRNAs was further assessed based on *in silico* prediction analysis.

### 2.3 Sample size estimation

In the current cohort, we collected samples from a total of 94 Pakistan women, including 64 patients diagnosed with PCOS and 30 healthy individuals from 30 families. The range of patient number for each family is 1-4 and the total number of patients is 64 from 30 families. To ensure that we have the control from each family, we recruited one healthy individual from each family.

### 2.4 Informed consent and recruitment of human subjects

The Rotterdam criteria have been applied for the diagnosis of individuals with PCOS. According to these criteria, a patient must exhibit two of the following three symptoms: hyperandrogenism (biochemical or clinical), oligo-ovulation or anovulation, and polycystic ovary morphology (PCOM), as determined through ultrasound examination. Following the acquisition of informed consent and a physical examination conducted by a gynecologist, selected patients underwent interviews regarding their family history and relevant details. Pedigrees were then constructed to trace the maternal inheritance pattern of this disorder.

Subsequently, saliva samples were collected from both PCOS-positive patients and healthy individuals. Exclusion criteria were applied to eliminate patients with hyperprolactinemia, thyroid and adrenal diseases, 21-hydroxylase deficiency, and androgen-secreting tumors, as these conditions can mimic the symptoms of PCOS. Ultimately, a total of 64 patients and 30 healthy individuals from 30 Pakistan families were selected for further analysis, focusing on the screening of mt-tRNA genes in maternally inherited PCOS patients. Variant calling and identification of both homoplasmic and heteroplasmic mutations were carried out as part of the subsequent analysis. Detailed family histories and information about deceased members were obtained, and pedigrees for these families were meticulously constructed.

### 2.5 DNA extraction, Sanger analysis and identification of variants

Saliva samples were subjected to DNA extraction utilizing the phenol-chloroform method as recently described (18). Quantification and assessment of DNA quality were conducted through Nanodrop quantification and gel electrophoresis. Primers were custom designed to amplify mt-tRNA genes. Subsequently, PCR was employed under specific conditions, and the resulting PCR products were submitted to a commercial company for DNA sequencing. Various online DNA analysis tools such as NCBI Blast (Basic Alignment Search Tool), Uniprot (Universal Protein Resource), and Unipro Ugene were utilized for additional alignment and investigations. In our study, we utilized the Revised Cambridge Reference Sequence (NC_012920.1) as the reference genome to identify mutations in our samples.

#### 2.5.1 QC value

The QC values of the mutation chromatograms in our samples exceed the recommended threshold of 40, with values ranging significantly higher, such as 81, 240, and beyond. These elevated QC values indicate the reliability and validity of the detected mutations.

### 2.6 Evaluation of pathogenicity of variants and validation by *in silico* analysis

MitoTIP (Mitochondrial tRNA Informatics Predictor) and PON-mt-tRNA, were applied for the identification and evaluation of pathogenicity of variations in mt-tRNA genes. Specifically, MitoTIP was utilized to determine the pathogenicity status of genetic variations whereas PON-mt-tRNA was employed to classify the variants based on multifactorial probability-based prediction (19–21). Structural analysis was conducted for mt-tRNAs using two web servers, RNA fold and R2dt.

## 3 Results

### 3.1 Clinical evaluations

The present investigation centered on the mt-tRNAs in individuals affected by maternally inherited PCOS. To accomplish this, a cohort of 64 PCOS patients and 30 healthy individuals from 30 Pakistani families, all with maternally inherited PCOS history, were chosen for the study. Additionally, the study concentrated on scrutinizing the mt-tRNA genes of these subjects to detect any mutations or genetic variations that might be linked to the onset of PCOS.

At the onset of the condition, individuals within the age bracket of 16-45 years were affected. These individuals presented with various health issues associated with PCOS. Among the observed problems were infertility and diabetes resulting from insulin resistance. Young females in the present investigation exhibited dermatological, metabolic, and hormonal challenges. Additionally, family members of these patients also experienced PCOS-related issues. Most symptoms, including dermatological obesity, hirsutism, menstrual irregularities, and hormonal imbalances, were found to be prevalent among a significant proportion of PCOS patients. **Table 1** summarizes the key clinical data of the PCOS patients and healthy control enrolled in this study, including age group, catamenia, infertility, dermatological issues, obesity, and diabetic problems. The detailed information of all clinical presentations, including hormonal profile (luteinizing hormone, LH; follicle-stimulating hormone, FSH; anti-Mullerian hormone, AMH), as well as free testosterone levels is shown in **Supplementary Table 1**.

**Table 1:** Summary of key clinical presentations of PCOS patients and healthy controls.

Clinical presentations		PCOS patients		Controls	
		No. of Cases	Percentage	No. of Cases	Percentage
Age group (years)	11-20	19	30%	6	20%
	21-30	23	36%	11	37%
	31-40	17	27%	10	33%
	41-50	5	8%	3	10%
	Total	64	100%	30	100%
Catamenia		49	77%	4	13%
Infertility		15	23%	3	10%
Dermatological issues		55	86%	1	3%
Obesity		27	42%	6	20%
Diabetes		15	23%	2	6%

### 3.2 Summary of mutations identified in mt-tRNA genes from the present study

**Figure 1:**
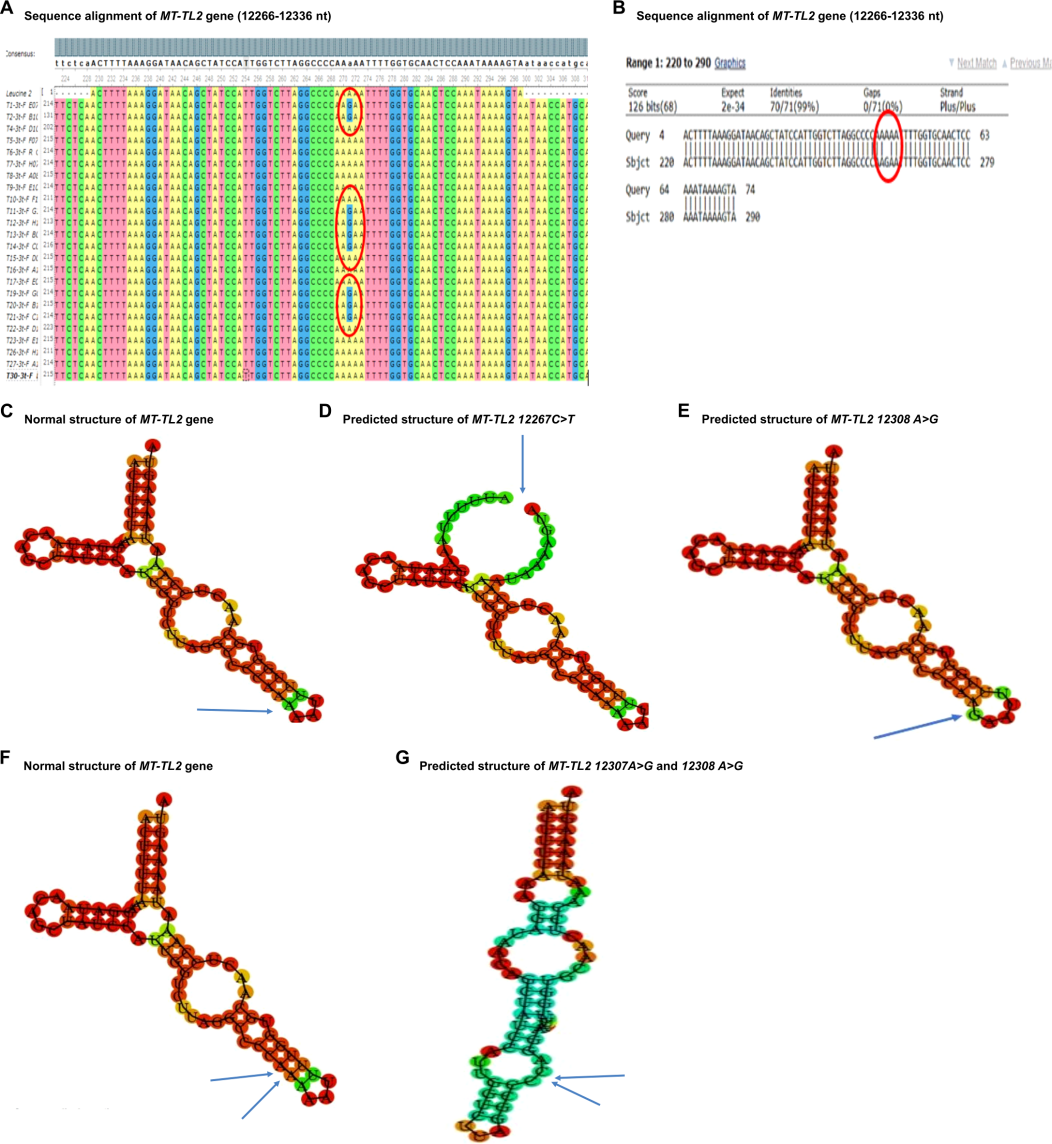
Sequence alignment and structural prediction of *MT-TL2* gene. **(A)** Sequence alignment of *MT-TL2* gene (12266-12336 nt) from maternally inherited PCOS-positive patients with rCRS Accession No. NC-012920.1 exhibiting A>G mutation at position 12307. **(B)** Sequence alignment of *MT-TL2* gene (12266-12336 nt) from maternally inherited PCOS-positive patients with rCRS Accession No. NC-012920.1 highlighting A>G mutation at position 12307. **(C)** Predicted structure of normal *MT-TL2* gene. **(D)** Predicted structure of *MT-TL2* gene carrying 12267 C>T variant. **(E)** Predicted structure of *MT-TL2* gene carrying 12308 A>G variant. **(F)** Predicted structure of normal *MT-TL2* gene. **(G)** Predicted structure of *MT-TL2* gene carrying 12307 A>G and 12308 A>G variant.

**Figure 2:**
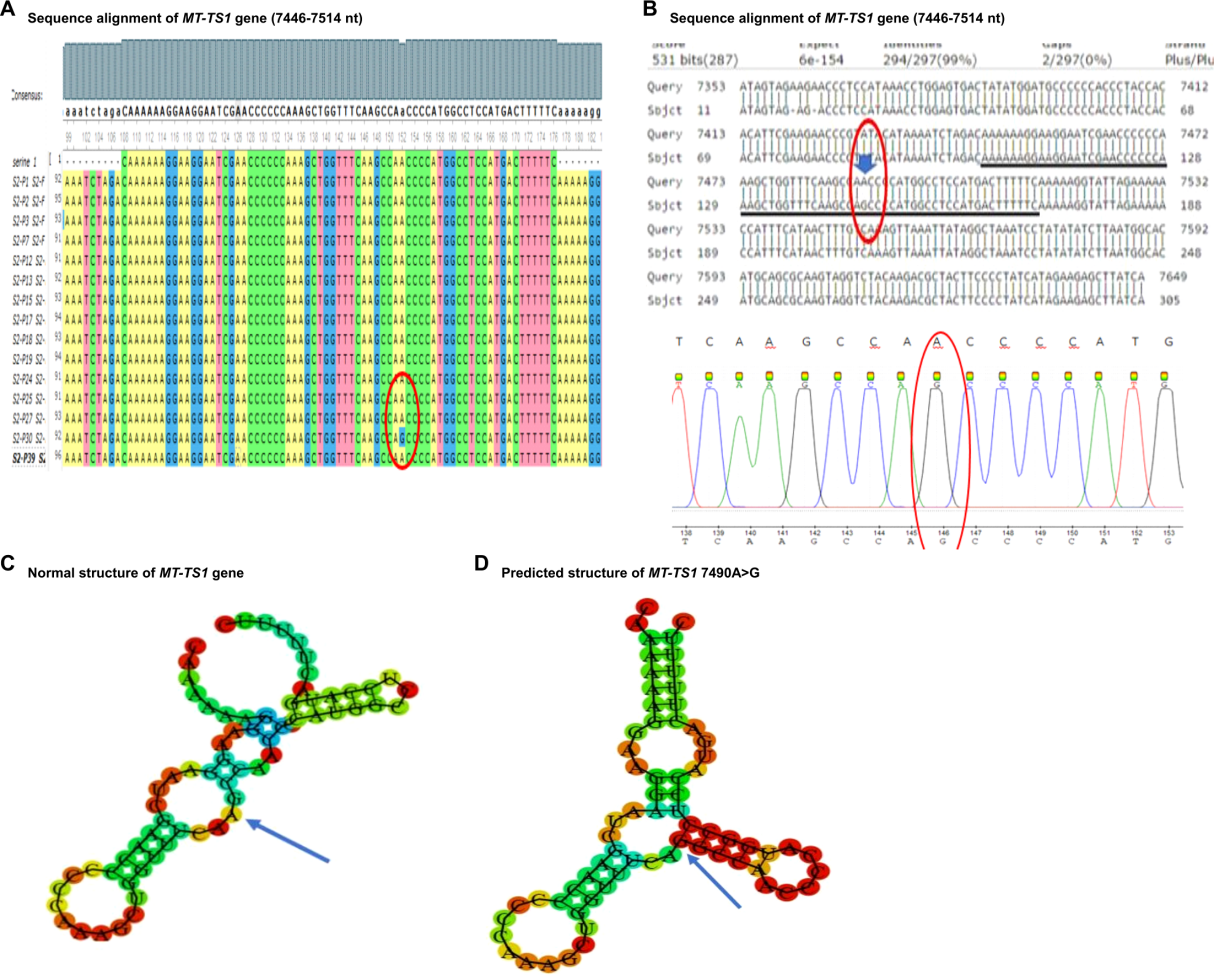
Sequence alignment and structural prediction of *MT-TS1* gene. **(A)** Sequence alignment of *MT-TS1* gene (7446-7514 nt) from maternally inherited PCOS-positive patients with rCRS Accession No. NC-012920.1 exhibiting A>G mutation at position 7490. **(B)** Sequence alignment of *MT-TS1* gene (7446-7514 nt) from maternally inherited PCOS-positive patients with rCRS Accession No. NC-012920.1 highlighting A>G mutation at position 7490. **(C)** Predicted structure of normal *MT-TS1* gene. **(D)** Predicted structure of *MT-TS1* gene carrying 7490 A>G variant.

**Figure 3:**
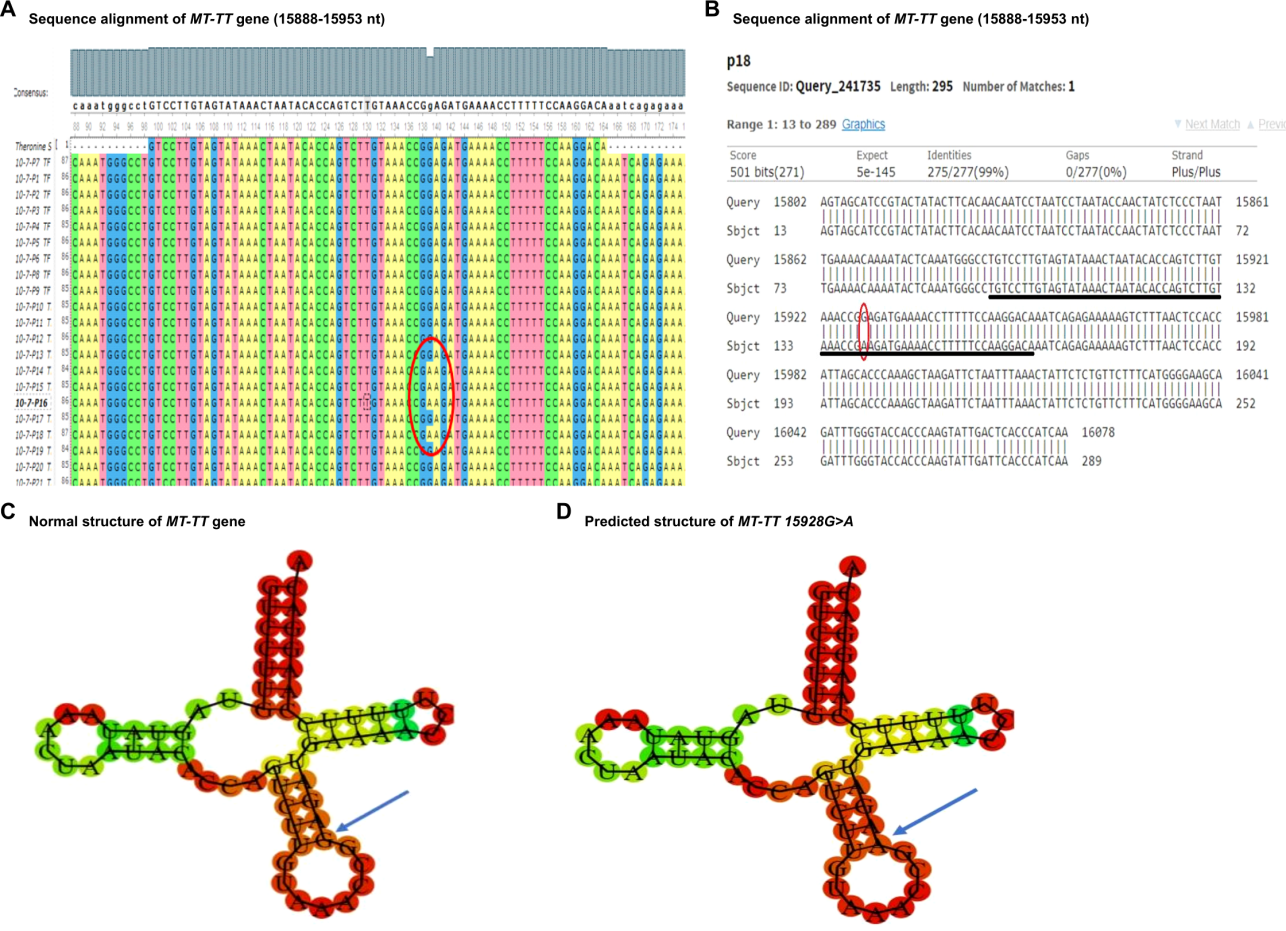
Sequence alignment and structural prediction of *MT-TT* gene. **(A)** Sequence alignment of *MT-TT* gene (15888-15953 nt) from maternally inherited PCOS-positive patients with rCRS Accession No. NC-012920.1 exhibiting G>A mutation at position 15928. **(B)** Sequence alignment of *MT-TT* gene (15888-15953 nt) from maternally inherited PCOS-positive patients with rCRS Accession No. NC-012920.1 highlighting G>A mutation at position 15928. **(C)** Predicted structure of normal *MT-TT* gene. **(D)** Predicted structure of *MT-TT* gene carrying 15928 G>A variant.

The nucleotide sequence analysis identified eight variants in five mt-tRNA genes including MT-TH, MT-TL2, MT-TS1, MT-TS2, and MT-TT genes. The Sanger sequence trace results, sequence alignment, and structural predictions of these variants are shown in **Figures 1**–**3**. We have observed the following features of these mutations. First, in addition to 12308 A>G in MT-TL2, the first variant in mt-tRNA gene we reported earlier in a Pakistani patient with PCOS (18), we observed two more variants, 12267 C>T and 12307 A>G in MT-TL2 gene, suggesting that these nucleotides may be essential for the function of MT-TL2 gene during the pathogenesis of PCOS. Second, all the other variants have not been previously documented in PCOS patients, but all of these mt-tRNA genes have been shown to carry variants in PCOS patients from different ethnic groups, including Chinese Han, Indian and Pakistani groups, suggesting that these five mt-tRNA genes may be the “hotspot” genes that have the high prevalence of mutations in PCOS patients across multiple ethnic groups. **Table 2** summarizes the comparison of all the variants identified in these five mt-tRNA genes across multiple ethnic groups.

**Table 2:** Comparison of variants of mt-tRNA genes identified in patients with PCOS across multiple ethnic groups.

No.	Allele identified in this study	Ethnic group in this study	Gene name	tRNA species	Variants previously reported	Ethnic group of patients previously reported	References
1	12179 A>T	Pakistan	MT-TH	tRNA^His (CAU/C)^	12161 T>G, 12172 A>G	Indian	(15)
2	12182 A>G						
3	12267 C>T	Pakistan	MT-TL2	tRNA^Leu (CUN)2^	12308 A>G	Pakistan	(18)
4	12307 A>G						
5	12308 A>G						
6	7490 A>G	Pakistan	MT-TS1	tRNA^Ser (UCN) 1^	7492 C>T	Chinese	(8)
7	12246 C>G	Pakistan	MT-TS2	tTRNA^Ser (AGU/C) 2^	12236 G>A	Indian	(15)
8	15928 G>A	Pakistan	MT-TT	tRNA^Thr (ACN)^	15914 A>T, 15924 A>G, 15930 G>A	Indian	(15)

**Figure 4:**
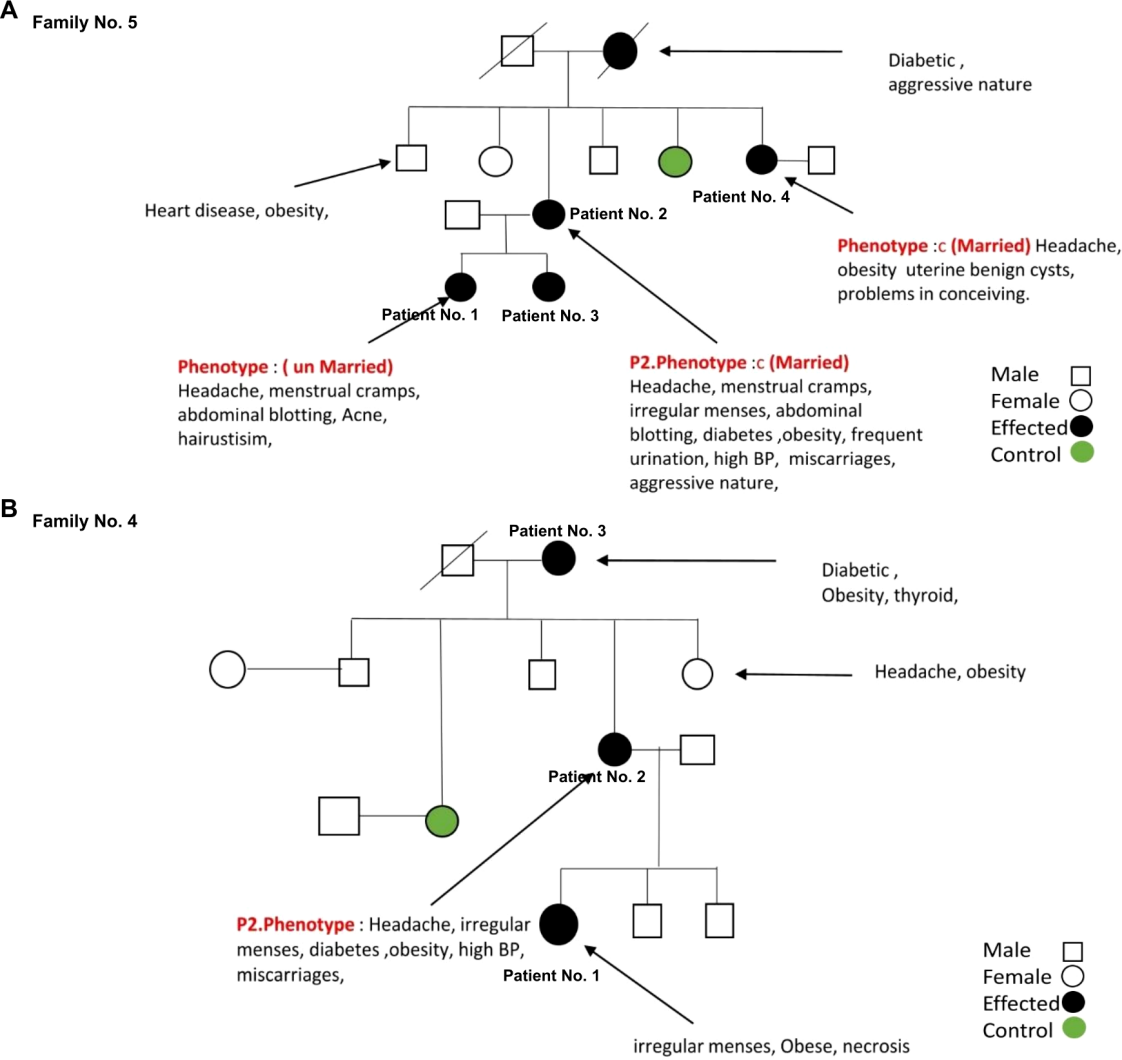
The family pedigree of family No. 5 **(A)** and No. 4 **(B)** based on the information provided.

Furthermore, in our cohort, we identified two patients with unique mutation patterns of mt-tRNA genes from families No. 4 and No. 5. In family No. 5, patient No. 2 carries two novel mutations of mt-tRNA genes: 12267 C>T in MT-TL2 and 12179 A>T in MT-TH, both of which have not been previously associated with any disease. In addition to all the clinical symptoms of PCOS, this patient experienced three miscarriages. Furthermore, the pedigree analysis showed that the family may share a similar health profile (**Figure 4A**). First, three more female family members are diagnosed with PCOS, including patient No. 4 (sister), and patient No. 1 and 3 (daughters). Second, this patient’s mother passed away due to diabetes and a heart attack. Third, this patient’s brother has been suffering with heart disease and obesity. In family No. 4, patient 2 presenting all the clinical symptoms of PCOS, carries 12246 C>G in MT-TS2 gene. Her family history indicated a predisposition to metabolic and endocrine disorders affecting multiple family members, including a diabetic and obese mother, patient 3, with thyroid disease, an obese sister with chronic headaches, a maternal aunt who died of breast cancer, and maternal cousins with obesity and PCOS-related symptoms (**Figure 4B**). Together, these results suggest a potential maternal inheritance pattern of PCOS and a broader familial predisposition to metabolic and cardiovascular complications.

### 3.3 Pathogenicity status of mt-tRNA variants

Next we evaluated the pathogenicity of the identified variants using multiple tools independently, including MitoTIP and PON-mt-tRNA. **Table 3** summarizes the pathogenicity status of the mutations revealed in this study. Although pathogenicity prediction of these mutations revealed minimal disruption or low impact as shown in MitoTIP Sub-Scoring as likely benign or possibly benign, some of the mutations identified in this study are associated with other diseases, such as breast cancer (12308 A>G in MT-TL2 and 15928 G>A in MT-TT) according to the Mito map allele search tool (26, 29). Importantly, given that these mutations are novel in the context of PCOS, it is plausible that these variants could potentially contribute to the development of these diseases, especially breast cancer in PCOS patients later in life, highlighting their relevance with and beyond PCOS and underscoring their potential long-term impact on patient health.

**Table 3:** Pathogenicity prediction of genetic variants detected in PCOS positive patients.

No	Gene name	Allele	MitoTIP Score	Comments	Reference
1	MT-TH	12179 A>T	25.30%	Possibly benign	(22)
		12182 A>G	46.40%	Possibly benign	(23)
2	MT-TL2	12267 C>T	65.2%	possibly pathogenic	(24)
		12307 A>G	44.70%	Possibly benign	(25)
		12308 A>G	42.00%	possibly benign	May increase the risk of stroke and breast cancer (26) and stroke (27)
3	MT-TS1	7490 A>G	1.40%	Likely benign	(28)
4	MT-TS2	12246 C>G	3.2%	likely benign	(28)
5	MT-TT	15928 G>A	20.20%	likely benign	May contribute to breast cancer (29) and bone metastasis (30)

## 4 Discussion

### 4.1 Mitochondrial mutations in PCOS

PCOS, the most common endocrine disorder affecting reproductive-age women globally, is the leading cause of infertility due to ovulatory dysfunction (1). Not surprisingly, PCOS presents as a highly heterogenic syndrome with variable manifestations in different ethnic and age groups (1). However, the biological basis for the phenotypic heterogeneity of PCOS remains not fully understood. A growing body of evidence suggests a clear link between mitochondrial dysfunction and PCOS. Mitochondria, the organelle essential for intracellular redox metabolism, produce ROS and release other intermediates normally neutralized by the antioxidant defense system inside the cells (6). To support this notion, mitochondrial abnormalities, including aberrant structure, biogenesis and activity of mitochondrial have been reported in PCOS patients (7). Furthermore, mutations in mtDNA have been identified in PCOS patients. These genetic variants in mitochondrial genomes may perturb the redox homeostasis and are thought to contribute to the clinical pathology of PCOS (6). Among the mtDNA mutations identified so far in PCOS patients, a large number of these mutations occur in mt-tRNA genes. It was proposed that mutations in mt-tRNA genes may disrupt the structure and functionality of mitochondrial RNA, causing the destabilization of the tertiary structure of mt-tRNAs and subsequent failure of mt-tRNA metabolism (7). Although the causative relationship between mutations in mt-tRNA genes and PCOS is still lacking, a recent transgenic mouse model provided compelling *in vivo* evidence demonstrating metabolic syndrome-like phenotypes in mice carrying a mutation orthologous to human pathological mutation in MT-TL1, thus shedding new molecular insights into mt-tRNA mutation-associated mitochondrial disorders, such as PCOS (16).

### 4.2 Mutations in common mt-tRNA genes identified in the current study

The present study aimed to investigate the new maternal genetic risk factors associated with PCOS by investigating the mt-tRNA genes in PCOS patients from Pakistan. Our findings identified seven novel mutations in five mt-tRNA genes from Pakistani PCOS patients. Interestingly, these mt-tRNA genes affected have been shown previously to harbor variants in PCOS women from distinct ethnic groups, including Chinese and Indian (**Table 2**).

The MT-TL2 gene is a small nucleotide RNA located on the P arm of mitochondrial DNA at position 12, spanning 75 base pairs. Up to now the pathological role of this variant in the function of MT-TL2 remains unclear. In our cohort, we have identified three mutations within this gene at distinct positions: 12267 C>T, 12307 A>G and 12308 A>G (**Figure 1**). Moreover, this gene has previously been documented in the Mito tip database and has been associated with various diseases, including CEPO, stroke, cardiomyopathy, breast cancer, renal cancer, prostate cancer, and alterations in brain pH leading to Creutzfeldt-Jakob disease (CJD). Furthermore, the mutation occurring at position 12267 C>T has not been documented on the Mito map, making it a novel finding in this context. Given that 12308 A>G variant has been identified in multiple independent cohorts of patients with PCOS (20 and the current study), the potential role of this mt-tRNA in the pathogenesis of PCOS warrants further investigation.

Among the mt-tRNA genes harboring mutations we have identified in the current study, other mutations on the same mt-tRNA genes have been reported from patients with PCOS, but from distinct ethnic groups. For example, MT-TS1 is a compact 69-nucleotide RNA molecule responsible for growing the serine amino acid to an elongating polypeptide chain at the ribosome location where protein synthesis occurs during translation. Our study identified a mutation at position 7490 A>G (**Figures 2A, B**). Furthermore, we conducted a thorough investigation into the mutation’s status using the Mito Map-Allele database to search for variants. To our knowledge, there is currently no report of this mutation on PON-mt-TRNAs specifically related to PCOS (**Table 3**). However, it’s worth noting that a recent study reported 7492 C>T mutation of the same gene in a Chinese PCOS patient (10). Given that the mutated nucleotides are highly conserved and are located in the anticodon stem of the MT-TS1 gene, these regions may play an important role for the steady-level, as well as the aminoacylation ability of tRNA. In line with the potential contribution of these mutations to the function of MT-TS1 gene, we utilized the RNA fold web server to generate structural models for the MT-TS1 gene. The normal MT-TS1 gene exhibited a minimum free energy of-10.80 kcal/mol, whereas the mutated structure displayed a slightly lower energy level of -13.90 kcal/mol, as shown **Figures 2C, D**, respectively.

In addition, the MT-TT gene, situated on the P arm of mitochondrial DNA at position 12 with a length of 66 base pairs, plays a crucial role in facilitating the incorporation of the amino acid threonine into the growing polypeptide chain at the ribosome site during protein synthesis. In our current study, we identified a specific mutation, positioned at 15928 G>A in PCOS patients. To evaluate the potential pathogenicity of this mutation, we utilized the Allele Search Tool within the Mito Map database and found that this mutation has been documented with a conservation rate of 48.89% (**Table 3**). Importantly, this alteration has been linked to various diseases, including multiple sclerosis and idiopathic recurrent miscarriages, and has also been catalogued in the ClinGen database. Interestingly a recent study revealed multiple variants in MT-TT gene in Indian patients with PCOS, including 15914 A>T, 15924 A>G and 15930 G>A (15). Furthermore, we performed structural prediction using the RNAFOLD web server. As shown in **Figures 3C, D**, both the reference and mutated structures show similar levels of the minimum free energy, suggesting that mutations at position 15928 G>A may not alter the structure of the MT-TT gene. Nevertheless, given the close proximity of the nucleotides carrying PCOS-associated variants across different ethnic groups, even though the contribution of MT-TT to the development of PCOS remains not fully understood, the potential pathological role of MT-TT in PCOS merits further studies.

Finally, MT-TH, a compact 69-nucleotide transfer RNA located in the human mitochondrial map region 12138–12206, facilitates the transfer of histidine amino acid to a developing polypeptide at the ribosomal site of protein synthesis. Our investigation revealed two mutations in MT-TH gene at distinct positions: the first at position 12179 A>T and the second at position 12182 A>G in PCOS patients. While the mutation at position 12179 A>T has not been previously documented, the mutation at position 12182 A>G has been reported in association with other diseases, albeit not linked to PCOS (**Table 3**). Notably, mutations of distinct nucleotides in MT-TH gene have been recently reported in POCS patients in South India, including 12161T>G and 12172A>G (15).

### 4.3 Significance of mt-tRNA mutations in different ethnic groups

Up to now, all the observed mutations in mt-tRNA genes in women with PCOS have originated from limited ethnic groups, particularly Chinese Han and South Indian (6–10, 12). Thus far, we have not seen any study in the literature in which multiple different ethnic groups were compared regarding the variants in mt-tRNA genes in PCOS patients. Given that the full spectrum of natural variations in mtDNA across wide populations is currently not available, we cannot rule out the possibility that there may be selection bias of patient samples that accounts for the observed mutations. Furthermore, due to the lack of cross-ethnic analysis of mt-tRNA genes in PCOS patients, it remains unclear whether there are any recurrent mutations in mt-tRNA genes potentially involved in the pathogenesis of PCOS.

To fill in this gap, in the current study, we have expanded our previous studies from three patients (18) to a cohort of 64 Pakistani women with PCOS. Importantly, the mt-tRNA genes affected have been shown previously to harbor variants in PCOS women from distinct ethnic groups (**Table 2**). To our knowledge, this is the first report comparing mutations of mt-tRNA genes in PCOS patients from different ethnic groups, such as Chinese Han, Indian and Pakistani women (**Table 2**). Based on our results, we did not observe any single mt-tRNA gene that harbors variations in all three ethnic groups. However, MT-TS1 appears to be the only mt-tRNA gene carrying variations between Chinese and Pakistan PCOS patients. Intriguingly, distinct variants were observed in three mt-tRNA genes, including MT-TH, MT-TS2 and MT-TT, that were shared between Indian and Pakistan PCOS patients. These data suggest that there are common mt-tRNA genes carrying PCOS-associated mutations that may be specific to certain ethnic populations. Further studies by including more ethnic groups and larger sample size are required to corroborate these findings and may help provide a full spectrum of variations in mt-tRNA genes across wide populations of PCOS. Together, our work provides new insights into the role of mt-tRNA genes in mitochondrial dysfunction underlying the pathophysiology of PCOS, highlighting mutations in mt-tRNA genes as potential important factors for future development of better diagnostic tools and more effective treatments for PCOS.

Furthermore, increasing evidence suggests that mitochondrial heteroplasmy, coexistence of wild-type and mutant mtDNA, may play an important role in PCOS (31). Given that most of variants of mt-tRNA identified so far occur in the evolutionarily conserved nucleotides, suggest that these variants may have the potential of damaging mt-tRNA structure, impairing tRNA processing, post-transcriptional modification, aminoacylation, or code recognition (32–34). Considering that ethnic-specific variants may exist in different ethnic groups of PCOS, it would be interesting to further investigate the potential role of mitochondrial heteroplasmy in the pathogenic functions of variants of mt-tRNA genes identified in this current study, as well as in other ethnic groups previously reported (6–10, 12, 18). In addition, given that the mutations of mt-tRNA genes may have the potential to cause biochemical defects of mitochondria due to a combination of factors, including alterations in the steady-state level of mt-tRNA, changes in tRNA structure, or the consequent aminoacylation possible due to the structural change, further studies are warranted to validate the functional roles of potential structure-disrupting variants identified in our screen.

Together, our findings suggest that there may be specific variations of mt-tRNA genes, or other mtDNA genes in PCOS women of different races and ethnicity. Furthermore, the pathogenicity status and structural prediction of the identified variants suggest the potential pathological role of mt-tRNA genes in PCOS (**Table 3**, **Figures 1**-**3**). Future studies to directly evaluate the function of these mt-tRNA genes will ascertain whether and how the mutations of mt-tRNA genes contribute to the development of PCOS. Overall, our results highlight mutations in mt-tRNA genes as important risk factors for further molecular diagnosis of PCOS, which may facilitate future development of better diagnostic tools and more effective therapies.

## Data Availability

The original contributions presented in the study are included in the article/**Supplementary Material**. Further inquiries can be directed to the corresponding authors.
