# Supplementary material for: Analysis of mutations in mitochondrial transfer RNA genes and the maternal inheritance of polycystic ovary syndrome
Source: Front Endocrinol (Lausanne). 2025 Feb 18;16:1509791. doi: 10.3389/fendo.2025.1509791 (PMC11876027; doi:10.3389/fendo.2025.1509791)
Supplement: Supplementary file 1 [file Table1.docx]

**Supplemental Table 1.**

**Detailed clinical presentations of PCOS patients and healthy controls from 30 families enrolled in the present study.**

| **Sample No.** | **Family No.** | **Cases** | **Age** | **Catamenia** | **Diabetes** | **Obesity** | **Infertility** | **Dermatological issues** | **Hormonal Profile** | | | **Free Testosterone level (nmol/L)** |
| --- | --- | --- | --- | --- | --- | --- | --- | --- | --- | --- | --- | --- |
|  |  |  |  |  |  |  |  |  | **LH (IU/L)** | **FSH (IU/L)** | **AMH (ng/mL)** |  |
| 1 | 1 | Patient 1 | 30 | - | - | - | - | + | 25.0 | 7.16 | 5.0 | 1.9 |
| 2 |  | Patient 2 | 33 | + | - | + | + | + | 20.0 | 7.0 | 8.0 | 2.02 |
| 3 |  | Control | 18 | - | - | - | - | - |  |  |  |  |
| 4 | 2 | Patient 1 | 30 | - | + | + | - | + | 18.0 | 6.0 | 7.25 | 2.0 |
| 5 |  | Patient 2 | 28 | + | - | - | - | + | 28.0 | 5.0 | 6.0 | 1.05 |
| 6 |  | Control | 35 | - | - | + | - | - |  |  |  |  |
| 7 | 3 | Patient 1 | 17 | + | - | + | - | + | 30.5 | 8.0 | 11.0 | 3.1 |
| 8 |  | Patient 2 | 23 | + | - | - | - | + | 25.0 | 5.0 | 4.55 | 2.00 |
| 9 |  | Control | 27 | - | - | - | - | - |  |  |  |  |
| 10 | 4 | Patient 1 | 18 | + | - | - | - | + | 40.67 | 7.0 | 11.0 | 2.8 |
| 11 |  | Patient 2 | 22 | + | - | - | - | + | 28.0 | 4.0 | 8.2 | 3.0 |
| 12 |  | Patient 3 | 44 | + | + | + | + | - | 32.0 | 3.76 | 6.0 | 2.6 |
| 13 |  | Control | 18 | - | - | - | - | - |  |  |  |  |
| 14 | 5 | Patient 1 | 26 | + | - | - | - | + | 24.0 | 4.0 | 7.20 | 1.9 |
| 15 |  | Patient 2 | 38 | + | - | - | + | + | 25.0 | 5.9 | 6.0 | 3.5 |
| 16 |  | Patient 3 | 21 | + | - | - | - | + | 39.0 | 5.5 | 4.4 | 1.9 |
| 17 |  | Patient 4 | 29 | - | - | + | - | + | 20.0 | 5.2 | 6.09 | 2.08 |
| 18 |  | Control | 28 | - | - | - | - | - |  |  |  |  |
| 19 | 6 | Patient 1 | 34 | + | - | + | - | + | 22.0 | 5.0 | 8.0 | 2.0 |
| 20 |  | Patient 2 | 19 | + | - | + | - | + | 33.0 | 5.0 | 5.0 | 1.9 |
| 21 |  | Control | 42 | - | - | - | - | - |  |  |  |  |
| 22 | 7 | Patient 1 | 20 | + | - | - | - | + | 25.0 | 4.9 | 6.0 | 2.0 |
| 23 |  | Patient 2 | 32 | + | - | - | - | + | 20.0 | 7.16 | 5.0 | 3.2 |
| 24 |  | Control | 22 | - | - | - | - | - |  |  |  |  |
| 25 | 8 | Patient 1 | 30 | - | + | + | - | + | 12.0 | 4.0 | 6.0 | 2.0 |
| 26 |  | Patient 2 | 42 | - | + | + | + | - | 22.0 | 6.0 | 6.0 | 3.5 |
| 27 |  | Control | 21 | - | - | - | - | - |  |  |  |  |
| 28 | 9 | Patient 1 | 18 | + | - | + | - | + | 30.0 | 5.0 | 4.9 | 2.00 |
| 29 |  | Patient 2 | 22 | + | - | - | - | + | 25.0 | 4.8 | 6.10 | 2.8 |
| 30 |  | Patient 3 | 33 | - | + | + | - | - | 40.67 | 4.0 | 10.0 | 2.1 |
| 31 |  | Control | 39 | - | - | - | + | - |  |  |  |  |
| 32 | 10 | Patient 1 | 25 | + | - | - | - | + | 28.0 | 7.0 | 8.20 | 3.0 |
| 33 |  | Patient 2 | 32 | + | - | + | - | + | 35.5 | 7.45 | 7.0 | 3.2 |
| 34 |  | Control | 25 | - | - | + | + | - |  |  |  |  |
| 35 | 11 | Patient 1 | 19 | + | - | - | - | + | 33.0 | 5.76 | 6.0 | 1.9 |
| 36 |  | Patient 2 | 34 | + | - | - | - | + | 25.25 | 4.0 | 4.4 | 3.02 |
| 37 |  | Control | 32 | - | - | - | - | - |  |  |  |  |
| 38 | 12 | Patient 1 | 18 | + | - | - | + | + | 28.0 | 8.16 | 6.0 | 1.9 |
| 39 |  | Patient 2 | 22 | + | - | - | - | + | 38.0 | 7.0 | 8.0 | 1.0 |
| 40 |  | Patient 3 | 36 | - | + | - | - | - | 18.0 | 5.0 | 7.25 | 2.9 |
| 41 |  | Control | 48 | - | + | - | - | - |  |  |  |  |
| 42 | 13 | Patient 1 | 36 | + | - | - | + | + | 29.0 | 7.0 | 6.0 | 3.0 |
| 43 |  | Patient 2 | 18 | + | - | - | - | + | 30.5 | 9.0 | 10.0 | 1.04 |
| 44 |  | Control | 32 | - | - | - | - | - |  |  |  |  |
| 45 | 14 | Patient 2 | 16 | + | - | + | + | + | 19.0 | 5.40 | 4.0 | 1.0 |
| 46 |  | Control | 18 | - | - | + | - | - |  |  |  |  |
| 47 | 15 | Patient 1 | 45 | - | + | + | - | + | 30.67 | 8.0 | 17.0 | 3.5 |
| 48 |  | Patient 2 | 28 | + | - | - | - | + | 39.0 | 9.0 | 8.20 | 2.0 |
| 49 |  | Control | 30 | - | - | + | - | - |  |  |  |  |
| 50 | 16 | Patient 1 | 27 | + | - | - | - | + | 32.0 | 3.76 | 6.0 | 2.4 |
| 51 |  | Patient 2 | 23 | + | - | - | - | + | 29.0 | 6.0 | 8.20 | 1.9 |
| 52 |  | Control | 22 | - | - | - | - | - |  |  |  |  |
| 53 | 17 | Patient 1 | 40 | + | + | + | + | - | 30.0 | 5.5 | 5.4 | 3.0 |
| 54 |  | Patient 2 | 18 | + | - | - | - | + | 20.0 | 5.20 | 6.09 | 1.8 |
| 55 |  | Control | 22 | + | - | - | + | + |  |  |  |  |
| 56 | 18 | Patient 1 | 34 | + | - | - | - | + | 32.0 | 5.0 | 9.0 | 2.8 |
| 57 |  | Patient 2 | 40 | - | - | + | - | + | 20.0 | 5.8 | 8.0 | 3.2 |
| 58 |  | Control | 34 | - | - | - | - | - |  |  |  |  |
| 59 | 19 | Patient 1 | 20 | + | - | + | - | + | 28.0 | 5.9 | 6.0 | 1.9 |
| 60 |  | Patient 2 | 19 | + | - | - | - | + | 27.0 | 5.16 | 5.02 | 1.7 |
| 61 |  | Control | 41 | - | + | - | - | - |  |  |  |  |
| 62 | 20 | Patient 1 | 29 | - | + | + | - | + | 12.0 | 4.0 | 6.09 | 1.9 |
| 63 |  | Patient 2 | 42 | - | + | + | + | - | 22.0 | 6.0 | 6.40 | 2.9 |
| 64 |  | Control | 18 | - | - | - | - | - |  |  |  |  |
| 65 | 21 | Patient 1 | 21 | + | - | - | - | + | 30.0 | 5.0 | 4.9 | 1.9 |
| 66 |  | Patient 2 | 26 | - | + | + | - | - | 25.0 | 4.8 | 6.10 | 1.60 |
| 67 |  | Control | 25 | - | - | - | - | - |  |  |  |  |
| 68 | 22 | Patient 1 | 32 | + | - | + | - | + | 40.67 | 4.0 | 10.0 | 2.0 |
| 69 |  | Patient 2 | 17 | + | - | - | - | + | 28.0 | 7.0 | 8.20 | 1.50 |
| 70 |  | Control | 34 | + | - | - | - | - |  |  |  |  |
| 71 | 23 | Patient 1 | 18 | + | - | - | + | + | 20.25 | 4.0 | 4.44 | 1.9 |
| 72 |  | Patient 2 | 22 | + | - | - | - | + | 25.0 | 3.76 | 6.0 | 1.8 |
| 73 |  | Control | 33 | - | - | - | - | - |  |  |  |  |
| 74 | 24 | Patient 1 | 33 | + | - | - | + | + | 25.0 | 7.16 | 5.0 | 2.5 |
| 75 |  | Patient 2 | 42 | - | + | + | + | - | 20.0 | 7.0 | 8.0 | 3.0 |
| 76 |  | Control | 18 | - | - | + | - | - |  |  |  |  |
| 77 | 25 | Patient 1 | 21 | + | - | - | - | + | 18.0 | 6.0 | 7.25 | 1.8 |
| 78 |  | Patient 2 | 26 | - | + | + | - | - | 28.0 | 5.0 | 6.0 | 2.8 |
| 79 |  | Control | 25 | - | - | - | - | + |  |  |  |  |
| 80 | 26 | Patient 1 | 32 | + | - | + | - | + | 30.5 | 8.0 | 11.0 | 2.8 |
| 81 |  | Patient 2 | 17 | + | - | - | - | + | 25.0 | 5.0 | 4.55 | 2.0 |
| 82 |  | Control | 34 | + | - | - | - | - |  |  |  |  |
| 83 | 27 | Patient 1 | 18 | + | - | - | + | + | 40.67 | 7.0 | 11.0 | 2.0 |
| 84 |  | Patient 2 | 22 | + | - | - | - | + | 18.0 | 4.0 | 8.2 | 3.9 |
| 85 |  | Control | 33 | - | - | - | - | - |  |  |  |  |
| 86 | 28 | Patient 1 | 33 | + | - | - | + | + | 39.0 | 3.76 | 6.10 | 3.8 |
| 87 |  | Patient 2 | 18 | + | - | + | - | + | 35.0 | 4.0 | 7.20 | 2.0 |
| 88 |  | Control | 21 | + | - | - | - | - |  |  |  |  |
| 89 | 29 | Patient 1 | 26 | - | + | + | - | - | 30.0 | 5.25 | 4.44 | 2.0 |
| 90 |  | Patient 2 | 25 | + | + | + | + | + | 29.0 | 5.20 | 6.09 | 2.9 |
| 91 |  | Control | 32 | - | - | + | - | - |  |  |  |  |
| 92 | 30 | Patient 1 | 17 | + | - | - | - | + | 22.50 | 5.0 | 8.0 | 3.0 |
| 93 |  | Patient 2 | 34 | + | - | - | - | + | 39.0 | 5.08 | 4.0 | 3.9 |
| 94 |  | Control | 18 | - | - | - | - | - |  |  |  |  |

**Note:** The presence of PCOS symptoms is indicated by the (+) sign in individuals diagnosed with PCOS. The absence of PCOS symptoms is denoted by the (-) in either PCOS patients or healthy controls. In each of 30 families, there is one healthy control individual. The number of PCOS patients in each family ranges from 1-4. The hormonal profile, including the levels of luteinizing hormone (LH, IU/L), follicle-stimulating hormone (FSH, IU/L), and anti-Mullerian hormone (AMH, ng/mL), as well as free testosterone levels (nmol/L) are indicated.
